# Glutamate gradually elevates [Zn^2+^]_i_ via the CaM–CaMKII–NOS cascade in primary cultured rat embryonic cortical neurons

**DOI:** 10.1038/s41598-025-99142-1

**Published:** 2025-04-30

**Authors:** Hui-Chiun Tseng, Yong-Sheng Wang, Chien-Yuan Pan

**Affiliations:** 1 Department of Life Science, National Taiwan University, 1 Roosevelt Rd. Sec 4, Taipei, 106 Taiwan; 2 Graduate Institute of Brain and Mind Science, National Taiwan University, Taipei, Taiwan

**Keywords:** Calmodulin, Calmodulin-dependent protein kinase II, Inflammation, Ionotropic glutamate receptor, Nitric oxide synthase, Zn^2+^, Cell biology, Neuroscience, Physiology

## Abstract

Zn^2+^ is essential for neuronal signaling, but imbalance cause cell death and neurodegenerative disorders. While the buffering system maintains low cytosolic Zn^2+^ concentration ([Zn^2+^]_i_), the details on physiological stimuli elevating [Zn^2+^]_i_ for neuronal processes remain limited. Our previous reports have demonstrated that dopamine elevates [Zn^2+^]_i_ through the cAMP−NO pathway, activating autophagy and inflammation in neurons. In this study, we adopted the Zn^2+^ imaging technique to verify how glutamate elevated [Zn^2+^]_i_ in cultured cortical neurons and examined the inflammatory response. Our results showed that glutamate elevates the [Zn^2+^]_i_, by activating ionotropic glutamate receptors. Inhibitors of calmodulin (CaM), CaM-dependent protein kinase II (CaMKII), and NO synthase (NOS) blocked the glutamate-induced Zn^2+^ response. High-K^+^ buffer induced-membrane depolarization significantly elevated the intracellular Ca^2+^ concentration ([Ca^2+^]_i_) but only slightly increased [Zn^2+^]_i_ and NO production. Glutamate also transiently increased NOS phosphorylation at Ser^1417^ within 15 min. The Zn^2+^ chelator, TPEN suppressed glutamate-induced inflammasome formation. These results indicate that glutamate-induced local increment in [Ca^2+^]_i_ via the ionotropic glutamate receptors activates the CaM−CaMKII−NOS complex to produce NO and elevate [Zn^2+^]_i_. which trigger inflammation in cultured neurons. Henceforth, this novel glutamate−Zn^2+^ signaling pathway after glutamate depolarization elevates [Ca^2+^]_i_ indicates the involvement of Zn^2+^ in modulating long-term neuronal activities.

**Supplementary Information:**

The online version contains supplementary material available at 10.1038/s41598-025-99142-1.

## Introduction

Zn^2+^ is an abundant trace metal ion in the body and presents in about 10% of human proteins as a structural or catalytic component^1^. Growing evidence supports the signaling roles of Zn^2+^ in various physiological cellular activities given the complex regulation system to maintain cytosolic Zn^2+^ homeostasis at pM–low nM level^2^. Many studies have associated Zn^2+^ with mental disorders and neurodegenerative diseases; however, most studies focused on the effects of insults, such as reactive oxygen species (ROS), cell damage, on elevating the intracellular Zn^2+^ concentration ([Zn^2+^]_i_)^3–5^. It is unclear that Zn^2+^ responses occur under physiological stimulations during neurotransmission in the neuronal circuit.

Three types of proteins are responsible for the intracellular Zn^2+^ homeostasis. At the membranes of cells and organelles, Zn^2+^ transporters (ZnTs) flux Zn^2+^ from the cytosol to the extracellular milieu and lumen of organelles; in contrast, Zrt- and Irt-like proteins (ZIPs) flux Zn^2+^ in the opposite direction to elevate the [Zn^2+^]_i_. In the cytosol, abundant metallothioneins (MTs, mw 5−8 kDa) contribute to buffering the Zn^2+^ elevation^2,6^. In contrast, ROS and nitric oxide (NO) interact with the thio groups of methionines in MTs, resulting in the dissociation of the bound Zn^2+^ to increase [Zn^2+^]_i_^7^. Accumulation of Zn^2+^ in the cell impairs mitochondria functions, enhances ROS production, activates NOS, and consumes more ATP, eventually leading to cell death^8,9^. ZnTs on the Golgi apparatus regulate the Zn^2+^ concentration in the lumen to modulate the activities of chaperon protein for functional protein secretion^10^. Overexpression of ZIPs at the ER membrane can rescue the vision caused by rhodopsin misfolding in drosophila^11^. Therefore, how these mechanisms regulate the Zn^2+^ homeostasis is important for various cellular activities.

Our previous reports reveal that dopamine activates the NO synthase (NOS) and generates NO to dissociate Zn^2+^ from MTs, resulting in an elevation in [Zn^2+^]_i_, which is necessary for the induction of autophagy and inflammation in cultured neurons^12,13^. Therefore, physiological stimulations that activate NO production may interfere with Zn^2+^ homeostasis. Glutamate is the most common excitatory neurotransmitter in the nervous system and could activate NOS to generate NO^14–16^. In this report, we loaded the primary cultured rat embryonic cortical neurons with a Zn^2+^ sensitive dye, FluoZin-3, to detect the changes in the [Zn^2+^]_i_. Upon glutamate stimulation, the [Zn^2+^]_i_ gradually elevated with a delay of 30 s and an EC_50_ of 7.9 ± 1.3 µM. With inhibitors against the downstream signaling pathway, we suggested that glutamate could elevated [Zn^2+^]_i_ via the calmodulin (CaM)−Ca^2+^/CaM-dependent protein kinase II (CaMKII)−NOS pathway. Moreover, this Zn^2+^ response was necessary for the glutamate-induced formation of inflammasomes in cultured neurons. In addition to inducing the electrical responses, glutamate may elevate [Zn^2+^]_i_ to initiate signaling pathways and regulate long-term neuronal activities.

## Results

### Glutamate elevates [Zn^2+^]_i_ dose-dependently in primary cultured cortical neurons

**Fig. 1:**
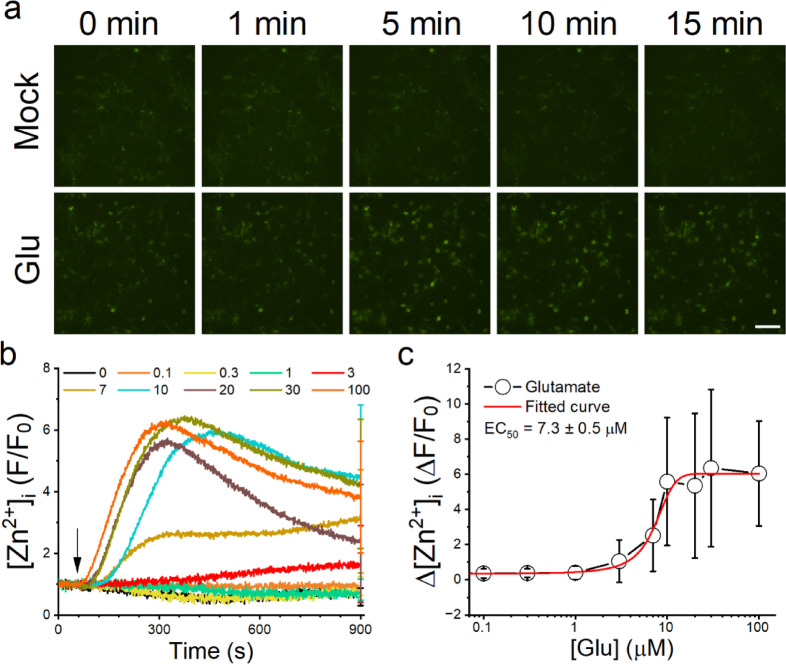
Glutamate elevates [Zn^2+^]_i_ dose-dependently in primary-cultured rat embryonic cortical neurons. We loaded the cultured neurons with FluoZin-3 and monitored the changes in fluorescence intensities. (**a**) Representative fluorescent images of neurons stimulated with HBSS containing glutamate (100 µM) or not (Mock). Images were taken at different times as indicated. Scale bar: 100 μm. (**b**) Representative average normalized fluorescence intensity traces stimulated with different concentrations of glutamate. F_0_ is the average fluorescence intensity before glutamate stimulation. Each trace represented the mean of 30 neurons from the identical batch of cultured neurons, with the SD displayed at the final recording point. The arrow indicates the application of glutamate, 1 min after the start of recording. (**c**) Representative dose-response curve of glutamate-induced changes in [Zn^2+^]_i_. The maximum changes of each neuron following glutamate stimulation (ΔF/F_0_) were averaged. Data are presented as Mean ± SD (*n* = 30 neurons for each dose in (**b**) from a single independent experiment and fitted using the Boltzmann equation.

Our prior study shows that dopamine elevates [Zn^2+^]_i_ via the cAMP−PKA−NOS signaling cascade^17^. Given that glutamate activates NOS via the NMDAR−CaMKII pathway, it is likely glutamate stimulation can elevate [Zn^2+^]_i_^18–20^. To confirm glutamate’s effect on [Zn^2+^]_i_ regulation in primary cultured rat embryonic cortical neurons, we loaded neurons with the Zn^2+^-sensitive fluorescent dye, FluoZin-3 and monitored fluorescence intensity changes upon glutamate stimulation (Fig. 1). Representative images showed an increase in fluorescence intensity in individual neurons within 15 min of glutamate addition (100 µM), while minimal changes were observed in the Mock group. Some neurons exhibited a continuous elevation throughout the recording period, whereas others reached a peak followed by a decline. After background subtraction, the fluorescence intensity before glutamate stimulation in each neuron was averaged as F_0_, and the traces were normalized to their respective F_0_ values (F/F_0_). To quantify the effect of glutamate on the Zn²⁺ response in individual neurons, we averaged the maximum changes (ΔF/F_0_) in normalized traces treated with different glutamate concentrations (Fig. S1).

To confirm the involvement of Zn^2+^, neurons were pretreated with TPEN (0.4 µM), a cell-permeable Zn^2+^ chelator, which attenuated the glutamate-induced increase in fluorescence intensity (Fig. S2). As shown in Fig. 1b, glutamate at concentrations below 1 µM did not significantly elevate the averaged F/F_0_ traces. However, at concentrations above 3 µM, fluorescence intensity increased in a dose-dependent manner. At concentrations below 7 µM, the averaged normalized fluorescence traces gradually increased until the end of the recording, whereas at 10 µM and above, the traces peaked and then declined. By averaging ΔF/F_0_ from neurons treated with different glutamate concentrations, we calculated an EC_50_ of 7.9 ± 1.3 µM (*N* = 3 independent neuronal culture batches), consistent with the binding affinities of ionotropic glutamate receptors^21^.

To determine whether this Zn^2+^ response occurs under physiological conditions, we performed whole-cell patch-clamp recordings in a neuron while simultaneously monitoring fluorescence intensities in nearby neurons (Fig. S3). Depolarizing the patched neuron with a 100-Hz stimulation (-70 to + 30 mV, 5 ms) for 1 min resulted in a pronounced increase in two nearby neurons, while others exhibited only a slight elevation. In contrast, when neurons were held at -70 mV without stimulation, no Zn²⁺ response was detected in nearby neurons (data not shown). These findings suggest that glutamate can elevate intracellular [Zn^2+^]_i_ in postsynaptic neurons, with the extent of elevation depending on the intensity of neurotransmission.

### Ionotropic GluR is responsible for the elevation of [Zn^2+^]_i_ in cultured neurons

**Fig. 2:**
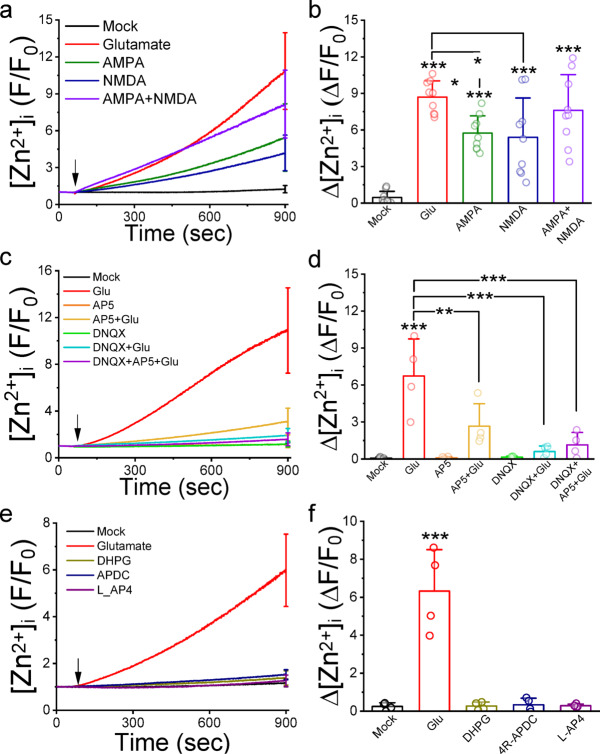
Agonists against iGluR elevate [Zn^2+^]_i_ in cultured cortical neurons. After loading the neurons with FluoZin3, we stimulated them with specific agonists and antagonists targeting various glutamate receptors. (**a**), (**c**) and (**e**) Representative average normalized fluorescence traces of neurons (F/F_0_). Each trace represented the mean of 20 neurons from the identical batch of cultured neurons, with the SD displayed at the final recording point. Arrows indicate the application of stimulants (1 min after the start of recordings). (**b**), (**d**), and (**f**) Average normalized changes (ΔF/F_0_). The chemicals used were glutamate (100 µM), DNQX (10 µM), AP5 (10 µM), DHPG (100 µM), (2R,4R)-APDC (100 µM), and L-AP4 (100 µM). N = 8, 4, & 4 for b, d, & f, respectively. N: number of independent neuron culture preparations for each group. **, and ***: one-way ANOVA with Tukey *post hoc* test with *p* < 0.01 and 0.001, respectively.

To discern which type of glutamate receptors (GluRs) is accountable for the glutamate-induced elevation of [Zn^2+^]_i_, we stimulated neurons with agonists and antagonists targeting ionotropic (iGluRs) and metabotropic (mGluRs) GluRs, observing changes in FluoZin-3 fluorescence intensity (Fig. 2). Glutamate (100 µM) significantly increased ΔF/F_0_ representing [Zn^2+^]_i_ changes from 0.46 ± 0.51 in the Mock group (*N* = 9 independent batches of neurons) to 8.70 ± 1.33 (*N* = 9, *p* = 1.05596E-9). Both AMPA (100 µM) and NMDA (100 µM) significantly elevated ΔF/F_0_ to 5.74 ± 1.43 (*N* = 9, *p* = 5.37055E-5) and 5.41 ± 3.20 (*N* = 9, *p* = 1.5233E-4), respectively. Co-application of AMPA and NMDA yielded ΔF/F_0_ of 7.62 ± 2.90, similar to glutamate-induced levels. Though slightly lower, AMPA and NMDA similarly raised [Ca^2+^]_i_ compared to glutamate (Fig. S4). To further validate iGluRs’ involvement, neurons were pretreated with AMPA receptor (AMPAR) and NMDA receptor (NMDAR) antagonists DNQX (10 µM) and AP5 (10 µM), respectively, before glutamate stimulation (Fig. 2c & d). Fluorescence traces showed both AP5 and DNQX mitigating glutamate-induced intensity increments. Glutamate-stimulated ΔF/F_0_ was reduced to 2.67 ± 1.81 (*N* = 4, *p* = 6.37349E-5) with DNQX and 0.61 ± 0.44 (*N* = 4, *p* = 0.00729) with AP5 pretreatment. Combined AP5 and DNQX reduced the response to 1.15 ± 1.00 (*N* = 4, *p* = 2.16934E-4). Examining mGluRs, specifically group I, II, and III, agonists DHPG (100 µM), (2R,4R)-APDC (100 µM), and L-AP4 (100 µM) respectively, did not significantly alter [Zn^2+^]_i_ (Fig. 2e,f). Glutamate significantly increased [Zn^2+^]_i_ from 0.25 ± 0.19 in the Mock group (*N* = 4) to 6.33 ± 2.18 (*N* = 4, *p* = 2.86737E-6), while mGluR agonists had no significant effect. Only DHPG (100 µM) raised [Ca^2+^]_i_ significantly to 1.19 ± 1.46 (*N* = 5, *p* = 0.0218) (Fig. S4d). These results affirm iGluRs as the primary glutamate-induced [Zn^2+^]_i_ elevation mediators.

### Inhibitors against CaM, CamKII, and nNOS suppress the glutamate-induced elevation of [Zn^2+^]_i_ in cultured neurons

**Fig. 3:**
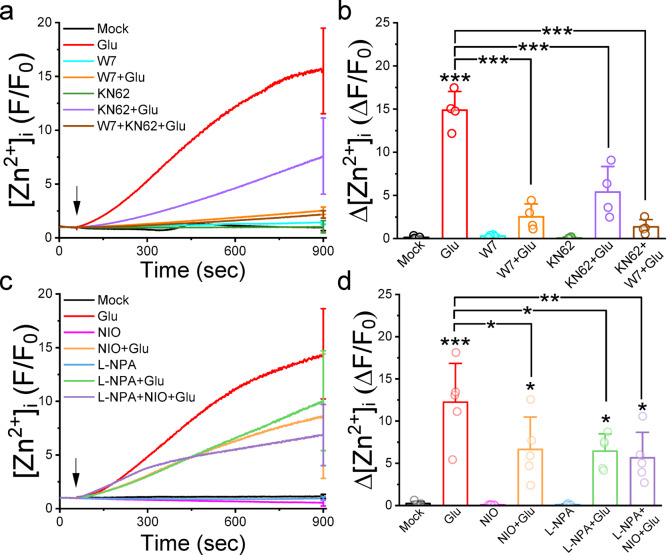
Antagonists against CaM, CaMKII, and nNOS block the glutamate-induced elevation of [Zn^2+^]_i_ in cultured cortical neurons. After loading neurons with FluoZin-3 in the presence of antagonists for 30 min, we stimulated neurons with glutamate (Glu, 100 µM) and recorded the fluorescence intensities in each single neuron. (**a**) and (**c**) Representative averaged normalized fluorescence traces (F/F_0_). Each trace represented the mean of 20 neurons from the identical batch of cultured neurons, with the SD displayed at the final recording point. Arrows indicate glutamate application (1 min after the start of recording). (**b**) and (**d**) Average changes in normalized fluorescence intensities (ΔF/F_0_). The chemicals used were W7 (50 µM), KN62 (10 µM), Vinyl-L-NIO (NIO, 1 µM), and L-NPA (NPA, 1 µM). Data presented were Mean ± SD from at least 4 independent neuron culture preparations and analyzed by one-way ANOVA with Tukey post hoc test, *, **, ***, indicate *p* < 0.05, *p* < 0.01, *p* < 0.001, respectively.

To validate the involvement of Ca^2+^-dependent activation of NOS activity in glutamate-induced [Zn^2+^]_i_ elevation, we applied inhibitors against CaM, CaMKII, and NOS (Fig. 3). Cultured neurons were pretreated with W7 (50 µM) and KN62 (10 µM), antagonists of CaM and CaMKII, respectively, while Vinyl-L-NIO (1 µM) and L-NPA (1 µM) were used to inhibit nNOS activity^22^. W7 and KN62 pretreatments significantly attenuated the glutamate-induced Zn^2+^ responses, averagely, from 14.87 ± 2.17 to 2.52 ± 1.51 (*N* = 4 independent batches of neurons, *p* = 5.13284E-8) and 5.39 ± 2.96 (*N* = 4, *p* = 4.1167E-7), respectively. Combined W7 and KN62 further reduced the Zn^2+^ response to 1.35 ± 0.85 (*N* = 4, *p* = 6.97171E-8). Both Vinyl-L-NIO and L-NPA significantly decreased glutamate-induced [Zn^2+^]_i_ elevation from 12.24 ± 4.59 to 6.65 ± 3.83 (*N* = 5, *p* = 0.03415) and 6.44 ± 2.04 (*N* = 5, *p* = 0.02558), respectively. These results suggest that the glutamate induced Zn^2+^ response involves the CaM, CaMKII, and NOS signaling pathways.

### Inhibiting CaM and CaMKII reduces the glutamate-induced generation of NO in cultured neurons

**Fig. 4:**
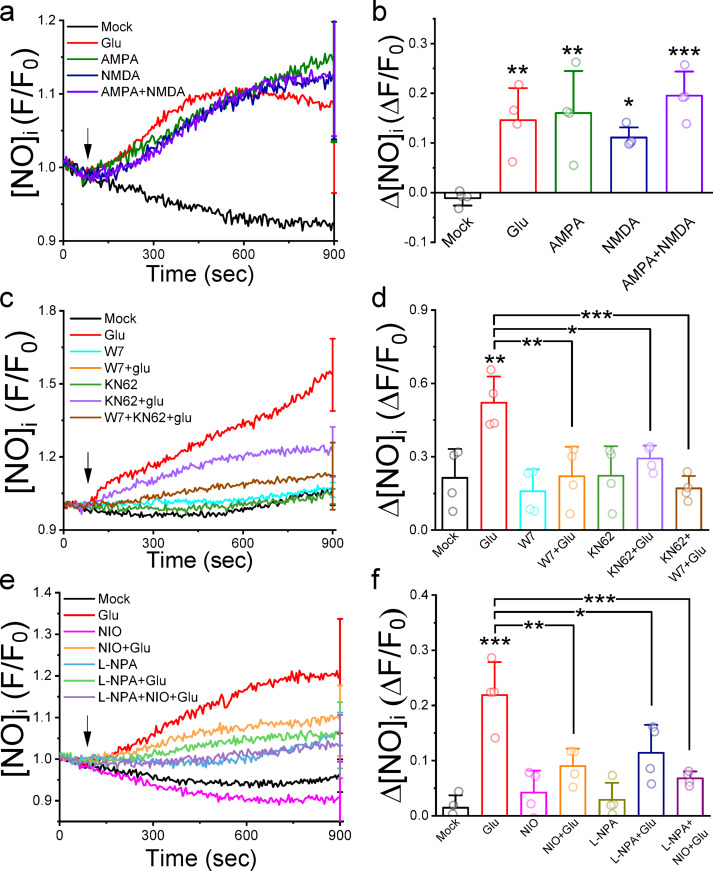
Glutamate-induced generation of NO is CaM- and CaMKII-dependent in primary-cultured cortical neurons. We loaded neurons with a NO-sensitive fluorescence dye, DAF-FM, and stimulated neurons with glutamate, AMPA, and NMDA (100 µM each), in the absence or presence of W7 (50 µM) and KN62 (10 µM), NIO (10 µM), and NPA (10 µM). (**a**), (**c**), and (**e**) Representative averaged normalized fluorescence traces (F/F_0_). Each trace represented the mean of 20 neurons from the identical batch of cultured neurons, with the SD displayed at the final recording point. Data are Mean ± SD from one batch of neurons with SD shown at the last time point. Arrows indicate the application of stimulants at 1 min after the start of recording. (**b**), (**d**), and (**f**) Average changes of normalized fluorescence response (ΔF/F_0_). Data presented were Mean ± SD from at least 4 independent neuron cultured preparations. batches of neurons, and the difference was analyzed with one-way ANOVA with Tukey post hoc test; *, **, and ***, indicating *p* < 0.05, 0.01, and 0.001, respectively, when compared to the Mock group.

To ascertain the involvement of CaM and CaMKII upstream of NOS activation, we loaded neurons with DAF-FM, a fluorescent NO-sensitive dye, and monitored for changes in fluorescence intensities under the presence of CaM and CaMKII inhibitors (Fig. 4). While the representative average normalized fluorescence traces (F/F_0_) of the Mock group declined gradually, glutamate, AMPA, and NMDA (100 µM each) applications increased F/F_0_ to similar levels. These significantly elevated averaged F/F_0_ from − 0.01 ± 0.02 of the Mock group (*N* = 4 independent batches of neurons) to 0.15 ± 0.06 (*N* = 4, *p* = 0.00666), 0.16 ± 0.08 (*N* = 4, *p* = 0.00319), and 0.11 ± 0.02 (*N* = 4, *p* = 0.03903), respectively. Pretreating neurons with W7 (50 µM) and KN62 (10 µM) effectively reduced glutamate-induced NO production, as the representative F/F_0_ traces show. The average ΔF/F_0_ revealed that W7 and KN62 significantly suppressed the glutamate-induced response from 0.52 ± 0.11 to 0.22 ± 0.06 (*N* = 4, *p* = 0.00473) and 0.29 ± 0.05 (*N* = 4, *p* = 0.04685), respectively. NIO and NPA, inhibitors of NOS, significantly decreased the glutamate-induced ΔF/F_0_ from 0.22 ± 0.06 to 0.09 ± 0.03 (*N* = 4, *p* = 0.0018) and 0.11 ± 0.05 (*N* = 4, *p* = 0.01343), respectively. TPEN pretreatment (0.4 µM) did not affect the glutamate-induced NO response (Fig. S5). These results support that glutamate stimulation activates NOS to synthesize NO via the CaM−CaMKII signaling cascade, resulting in [Zn^2+^]_i_ elevation.

### Direct membrane depolarization only slightly elevated [Zn^2+^]_i_ in cultured neurons

**Fig. 5:**
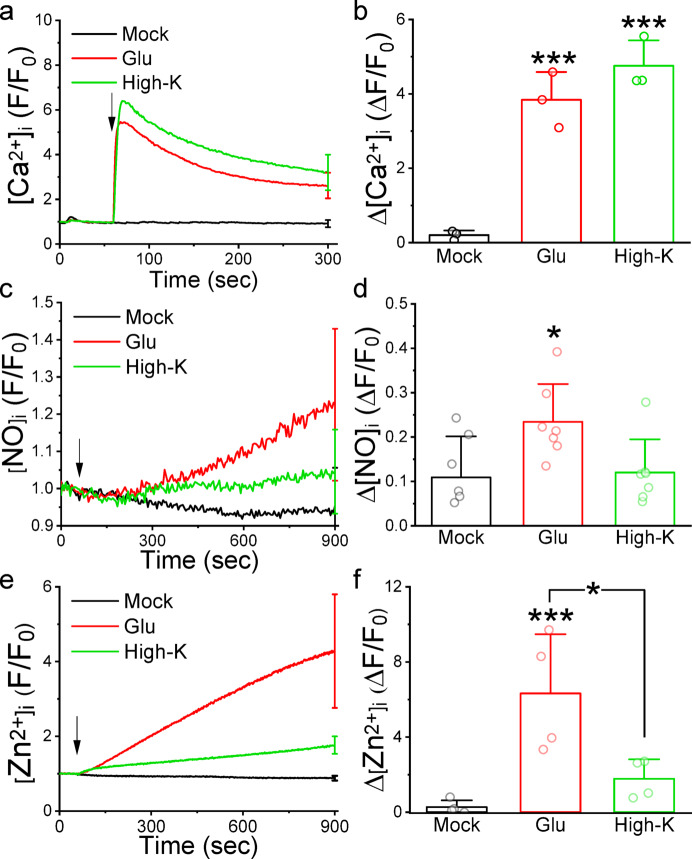
High-K^+^ stimulation did not increase the cytosolic NO and Zn^2+^ in cultured cortical neurons. We loaded the neurons with Fluo-2, DAF-FM, and FluoZin-3 to monitor the changes in the cytosolic concentrations of Ca^2+^ (**a** and **b**), NO (**c** and **d**), and Zn^2+^ (**e** and **f**), respectively. By adding a High-K^+^ buffer at a 1:1 ratio, we increased the concentration of K^+^ in the bath to 70 mM and recorded the neurons’ fluorescence intensities. (a), (c), & (e) Representative averaged normalized traces (F/F_0_). Each trace represented the mean of 20 neurons from the identical batch of cultured neurons, with the SD displayed at the final recording point. Data were averages from one batch of neurons, and SD was displayed at the last recording point. Arrows indicate the application of stimulants at 1 min after the start of recording. (**b**), (**d**), and (**f**) Average changes in the normalized fluorescence intensity (ΔF/F_0_). Data presented were Mean ± SD from 3, 4, and 7 independent neuron culture preparations, respectively. The statistical differences were analyzed with one-way ANOVA and Tukey post hoc test; * and *** indicate *p* < 0.05 and 0.001, respectively.

Membrane depolarization triggers voltage-gated Ca^2+^ channels (VGCC), leading to Ca^2+^ influx and elevated [Ca^2+^]_i_, potentially initiating the CaM−CaMKII signaling pathway^23^. To assess if VGCC-mediated Ca^2+^ influx is implicated in glutamate-induced Zn^2+^ response, we depolarized cells using a High-K^+^ buffer and monitored fluorescence signals from neurons stained with Fluo-2, DAF-FM and FluoZin-3 independently (Fig. 5). Normalized fluorescence traces revealed that High-K^+^ stimulation promptly elicited a Ca^2+^ response slightly exceeding that induced by glutamate. However, High-K^+^ stimulation did not elevate NO production and [Zn^2+^]_i_ to the extent observed with glutamate. Both High-K^+^ and glutamate significantly increased [Ca^2+^]_i_ from 0.20 ± 0.12 of the Mock group to 3.84 ± 0.75 (*N* = 3 independent batches of neurons, *p* = 1.95239E-4) and 4.76 ± 0.68 (*N* = 3, *p* = 6.8125E-4), respectively. Glutamate significantly augmented NO production and [Zn^2+^]_i_ from 0.11 ± 0.09 and 0.27 ± 0.36 of the Mock group to 0.23 ± 0.09 (*N* = 4, *p* = 0.03202) and 6.32 ± 3.15 (*N* = 7, *p* = 0.00412), respectively. In contrast, High-K^+^ only slightly increased NO and [Zn^2+^]_i_ to 0.12 ± 0.02 (*N* = 4, *p* = 0.96696) and 1.78 ± 1.03 (*N* = 7, *p* = 0.53162), respectively, with no significant difference from the Mock group. While Ca^2+^ is crucial for activating CaM−CaMKII signaling, our findings suggest that the type of ionic channels mediating Ca^2+^ influx influences the ability to activate the CaM−CaMKII−nNOS signaling cascade and elevate [Zn^2+^]_i_.

### Glutamate transiently increases the phosphorylation at Ser^1417^ of nNOS in cultured neurons

**Fig. 6:**
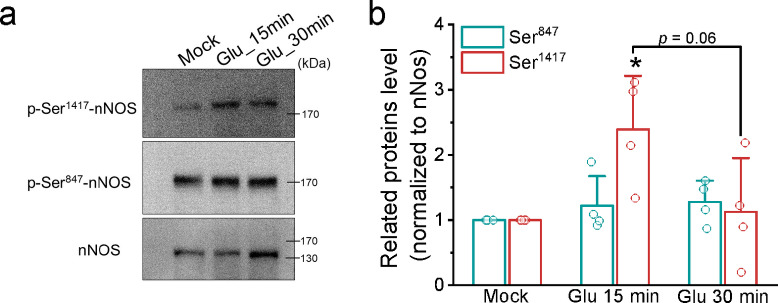
Glutamate treatment transiently phosphorylates nNOS at Ser^1417^ in cultured neurons. We stimulated the cultured neurons with glutamate (100 µM), then harvested the total proteins at 15 and 30 min for Western blot analysis with specific antibodies against nNOS, Ser^847^-nNOS, and Ser^1417^-nNOS. We then normalized the band intensities of these phosphorylated nNOS to each experiment’s corresponding nNOS and Mock group. (**a**) Representative immunoblots. (**b**) Normalized staining levels. Data presented were Mean ± SD from at least 4 independent neuron culture preparations, with statistical significance assessed via one-way ANOVA and Tukey post hoc test (*: *p* < 0.05).

Several studies have suggested that CaMKII phosphorylates nNOS at Ser^847^ and Ser^1417^ (equivalent to human Ser^1412^), leading to decreased and increased NO synthesis activity, respectively^14–16^. To investigate how glutamate stimulation affects nNOS phosphorylation levels in cultured neurons, we conducted Western blot analysis using specific antibodies against these phosphorylation sites (Fig. 6). The representative blotting images demonstrate that glutamate (100 µM) did not alter the Ser^847^ phosphorylation level at 30 min; however, phosphorylation at Ser^1417^ increased at 15 min and then declined by 30 min. After normalization to the total amount of nNOS, glutamate treatment significantly elevated the phosphorylation level at Ser^1417^ to 2.39 ± 0.82 (*N* = 4 independent batches of neurons, *p* = 0.04066) at 15 min, followed by a decrease to 1.13 ± 0.83 (*N* = 4, *p* = 0.96251) at 30 min. There was no significant difference in the phosphorylation level at Ser^847^ after glutamate treatment. High-K^+^ stimulation had no significant effect on nNOS phosphorylation at Ser^847^ after 30 min; in contrast, Ser^1417^ phosphorylation remained unchanged at 15 min but increased at 30 min (Fig. S6). These findings suggest that glutamate transiently activates nNOS by phosphorylating Ser^1417^, leading to increase NO production, likely contributing to the Zn^2+^ response.

### Glutamate-induced formation of NLRP3-inflammasomes is Zn^2+^-dependent in cultured cortical neurons

**Fig. 7:**
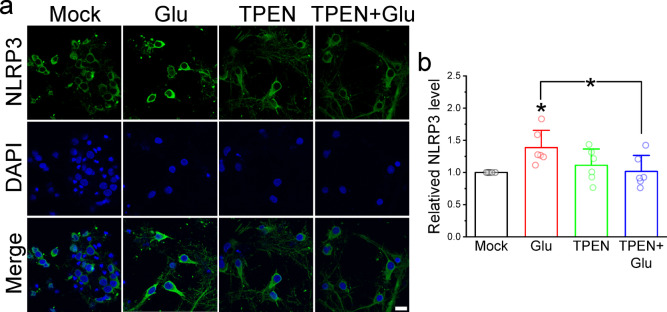
Glutamate increases the immunostaining level of NLRP3 in cultured cortical neurons. We treated the neurons with glutamate (100 µM) in the absence and presence of TPEN (0.4 µM) for 30 min. Subsequently, the neurons were immuno-stained with an antibody against NLRP3 and DAPI to visualize the localization of inflammasomes and nucleus, respectively. (**a**) Representative confocal fluorescence images of neurons. Scale bar: 10 μm. (**b**) Normalized average fluorescence intensity of stained NLRP3. We randomly chose 20 neurons from each treatment group for fluorescence intensity quantitation and normalized against the Mock group. Data represent the Mean ± SD from 6 independent neuron culture preparations, with statistical significance assessed via one-way ANOVA and Tukey *post hoc* test (*: *p* < 0.05).

Our prior study established the Zn^2+^ dependency of dopamine-induced inflammation in cultured cortical neurons^13^. To explore whether a similar Zn^2+^-dependent mechanism underlies glutamate-induced inflammation, we exposed neurons to glutamate for 30 min, with or without TPEN (0.4 µM), followed by immunostaining for NLRP3, an inflammasome marker (Fig. 7). Immunofluorescence images demonstrated that glutamate increased NLRP3 immunofluorescence, which TPEN pretreatment attenuated. Normalized fluorescence intensities revealed a significant elevation in cytosolic NLRP3 levels induced by glutamate (1.38 ± 0.27, *N* = 6 independent batches of neurons, *p* = 0.03246) compared to the Mock group. TPEN alone did not affect staining levels (1.11 ± 0.25, *N* = 6, *p* = 0.17516) but significantly suppressed the glutamate-induced increase to 1.02 ± 0.25 (*N* = 6, *p* = 0.04204).

Given the potential for glutamate-induced excitotoxicity to cause mitochondrial damage and inflammation^24,25^, we assessed cell viability in neurons treated with glutamate for different time periods (Fig. S7 and S8). A recent report reveals that treating cultured cortical neurons with 100 µM glutamate for 6 h does not significantly reduce cell viability^26^. Similarly, our results showed no significant viability reduction, with or without TPEN pretreatment, after 15–30 min of exposure to 100 µM glutamate (Fig. S7). After 6 h, 30 µM glutamate had no effect, while 100 µM caused a slight but non-significant reduction in viability. However, after 18 h, both 30 µM and 100 µM glutamate significantly reduced cell viability by approximately 50% (Fig S8). These findings indicate that glutamate-induced excitotoxicity is unlikely to affect cell viability during the 15-minute imaging period, as its toxic effects require prolonged exposure. Furthermore, they support the notion that a Zn²⁺-dependent inflammatory response can be initiated in cultured neurons following glutamate treatment without significant mitochondrial damage.

## Discussion

To ascertain the role of Zn^2+^ as a signaling molecule in regulating cellular activities, it is essential to identify the physiological stimuli responsible for increasing [Zn^2+^]_i_ to activate Zn^2+^-responsive proteins and pathways^27–29^. Our previous findings have established that dopamine raises [Zn^2+^]_i_ through the cAMP−PKA−NOS pathway to activate autophagy and inflammation in cultured neurons^13,17^. Building upon this, we unveil that glutamate, the principal excitatory neurotransmitter, triggers a localized surge in [Ca^2+^]_i_ via the NMDAR, initiating the CaM−CaMKII−NOS signaling cascade. This leads to NO synthesis, which liberates Zn^2+^ from metallothioneins, gradually increasing [Zn^2+^]_i_ and activating inflammation. Consequently, glutamate sequentially depolarizes the membrane potential, elevates [Ca^2+^]_i_, and subsequently increases [Zn^2+^]_i_ during neurotransmission. The involvement of Zn^2+^ introduces a fresh perspective in investigating long-term circuit plasticity.

In contrast to the immediate elevation observed in [Ca^2+^]_i_, glutamate induces a gradual increase in [Zn^2+^]_i_ with a delay of approximately 20–30 s. The dose-response curve exhibits an EC_50_ of 7.9 ± 1.3 µM (*N* = 3) and demonstrates a pronounced rise in Zn^2+^ response within the 3 to 10 µM glutamate concentration range (Fig. 1). Considering the concentration of glutamate in the synaptic cleft and extrasynaptic region, which could reach mM and hundred µM levels, respectively^30^, and the binding affinities of iGluRs for glutamate, typically ranging between 1 and 10 µM^21^, it is evident that glutamate can effectively elevate [Zn^2+^]_i_ via iGluRs during neurotransmission. The observed delay in each single neuron and sharp increase in the dose-response curve imply the involvement of signaling molecule accumulation over time. In addition, following the immediate membrane depolarization and elevation in [Ca^2+^]_i_ induced by glutamate, the persistent presence of glutamate in the synapse leads to the accumulation of signaling molecules, eventually triggering the elevation of [Zn^2+^]_i_.

Several reports have highlighted various mechanisms through which glutamate can elevate Zn^2+^ levels. GluR2-lacking AMPARs have been identified as permeable to Zn^2+^, allowing its influx into the cytosol^31,32^. Additionally, studies suggest that glutamate stimulation may activate ROS production, leading to Zn^2+^ release from mitochondria and cytosolic Zn^2+^-binding proteins in rat forebrain neurons^4^. In hippocampal neurons, ATP consumption by Ca^2+^ pumps to buffer elevated [Ca^2+^]_i_ results in cytosolic acidification, leading to the dissociation of Zn^2+^ from MTs^19^. Our research indicates that while High-K^+^ buffer stimulation dramatically elevates [Ca^2+^]_i_, it only slightly increases [Zn^2+^]_i_ (Fig. 5), suggesting that Ca^2+^ elevation may not be the primary factor responsible for glutamate-induced Zn^2+^ elevation. Moreover, blocking Zn^2+^ elevation with TPEN does not affect NO production (Fig. S4). In contrast, suppressing NO production inhibits glutamate-induced Zn^2+^ elevation (Fig. 3), implying that NO acts upstream of Zn^2+^ in the signal transduction pathway.

At the postsynaptic synapse, NMDAR, CaM, CaMKII, and nNOS are colocalized, with CaM acting as the mobile factor in activating CaMKII and nNOS^20^. The influx of Ca^2+^ via NMDAR initiates a local elevation of [Ca^2+^]_i_ and activates CaM. However, High-K^+^ stimulation triggers a global elevation in [Ca^2+^]_i_, which is higher than that induced by glutamate due to the activation of VGCCs. Despite this, the Ca^2+^ influx from High-K^+^ stimulation is not efficient in activating CaM and only slightly elevates intracellular [Zn^2+^]_i_ (Fig. 5). Additionally, neither the Ca^2+^ release from intracellular Ca^2+^ stores via the activation of type I mGluRs significantly elevates [Zn^2+^]_i_. Therefore, Ca^2+^ is necessary for the glutamate-induced Zn^2+^ response, and crucial is the pathway through which Ca^2+^ enters the cell.

Our findings indicate that the activation or blocking of either AMPAR or NMDAR yields similar effects in elevating [Ca^2+^]_i_, [Zn^2+^]_i_, and NO production, although at lower levels induced by glutamate. AMPAR lacking the GluA2 subunit is permeable to both Ca^2+^ and Zn^2+^, ^33^. Similarly, channels with Ca^2+^ permeability, such as NMDAR and VGCCs, may also be permeable to Zn^2+^, ^18^. However, it is unlikely that Zn^2+^ flux into the neurons via these membrane ion channels, as NO production is a prerequisite for the iGluR-induced elevation of [Zn^2+^]_i_.

The phosphorylation status of nNOS at specific sites is pivotal in regulating its activity. Phosphorylation at Ser^847^ decreases NO synthase activity and increases superoxide generation, whereas phosphorylation at Ser^1417^ enhances NO generation activity^34^. Our results demonstrate a transient increase in the phosphorylation level of Ser^1417^ within 15 min, followed by a decline to resting levels within 30 min, with no observable change in the immunostaining of Ser^847^. Additionally, blocking the activities of CaMKII and nNOS suppresses the glutamate-induced elevation of [Zn^2+^]_i_. These findings support that glutamate activates CaMKII to phosphorylate nNOS at Ser^1417^, generating NO for the Zn^2+^ response.

MTs are well-known targets of NO, and nitrosylation leads to the dissociation of bound Zn²⁺ from MTs into the cytosol, thus increasing the [Zn^2+^]_i_^17,35^. However, the formation of peroxynitrite from the interaction between NO and superoxide interferes with mitochondrial respiration and releases Zn²⁺ from intracellular stores^36^. Our results from the cell viability MTT assay (Fig. S7 & S8), which monitors mitochondrial function, suggest that glutamate does not cause significant damage to the mitochondria during the fluorescence recording. Therefore, it is unlikely that Zn^2+^ released from intracellular stores is responsible for the glutamate-induced elevation of [Zn^2+^]_i_.

While most studies have focused on NO’s modifications of various proteins through nitrosylation or nitration reactions, our research demonstrates that NO can lead to the elevation of [Zn^2+^]_i_. This elevation of [Zn^2+^]_i_ serves as a significant factor in activating the inflammatory response in cultured neurons (Fig. 7). Neuroinflammation has garnered considerable attention due to its involvement in the early stages of neurodegenerative diseases and mental disorders^37^. Contrary to traditional views, inflammation may benefit the CNS by promoting axon growth, neurogenesis, and protection^38^. However, the precise impact of Zn^2+^-induced inflammation on neurodegeneration requires further investigation for a comprehensive understanding.

Depending on the firing pattern and intensity of neurotransmission, neurotransmitters activate various signaling pathways to regulate synaptic plasticity^39^. Our research demonstrates that in addition to depolarizing the membrane potential and increasing [Ca^2+^]_i_, glutamate can activate the CaM–CaMKII–nNOS pathway to elevate [Zn^2+^]_i_. This delayed Zn^2+^ response suggests that robust and sustained neurotransmission accumulates sufficient signaling molecules, enabling the dissociation of Zn^2+^ from binding proteins and activation of Zn^2+^-dependent pathways. Thus, our findings unveil a novel Zn^2+^ signaling transduction pathway during neurotransmission in neurons, highlighting the complexity and versatility of synaptic regulation mechanisms.

## Methods

### Chemicals

Dulbecco’s Modified Eagle’s medium, FluoZin-3 AM, Fluo-2 AM, DAF-FM, and 3-(4,5-dimethylthiazol-2-yl)-2,5-diphenylterazolium bromide (MTT) were purchased from Life Technology Inc. (Carlsbad, CA, USA). Vinyl-L-NIO (NIO), Nω-Propyl-L-arginine hydrochloride (L-NPA), and W-7 hydrochloride (W7) were purchased from Cayman Chemical (Ann Arbor, MI, USA). Unless otherwise indicated, all other chemicals were purchased from Sigma-Aldrich, Inc. (St. Louis, MO, USA).

### Primary culture of rat embryonic cortical neurons

The Sprague-Dawley rat embryos were obtained from pregnant rats at embryonic day 14.5 (E14.5), purchased from BioLasco Co. (Taiwan). We confirm that all experiments were performed in accordance with relevant IACUC and ARRIVE guidelines and regulations. We totally sacrificed 20 pregnant rats with an average of 15 embryos per dam biweekly for this report. For each batch of cultured neurons, a pregnant rat was terminally anesthetized with tiletamine-zolazepam (50 mg/kg, i. p.; Zoletil, Vibac, France) with xylazine (10 mg/kg, Rompun, Bayer, Germany). Abdominal skin was washed with 70% ethanol followed by an incision to cut and open the peritoneal cavity. Amniotic sacs were exposed with scissors and an average of 15 embryos per dam were dissected out from the uterus. The dam was then euthanized via cardiac puncture while still under anesthesia using a 23G needle, ensuring the cessation of heart activity was confirmed. Those embryos were subsequently transferred to a 10-cm cultured dish containing 30 ml of Ca^2+^/Mg^2+^-free Hank’s buffer salt solution (HBSS) placed on ice, and moved to a sterile laminar hood for further processing. The cortical neurons were isolated and cultured from the forebrains of those embryos with the protocol described before^40^. The cortical neurons from all of the embryos of either sex were pooled together for culture. This procedure complies with the Animal Welfare Regulations and is approved by the Institutional Animal Care and Use Committee of National Taiwan University (Permit No. 110-00005). Neurons were cultured in a Neuro-basal medium supplemented with B27 (Thermo Fisher Inc., Waltham, USA) in humidified air at 37 ℃ with 5% CO_2_. All experiments were operated with neurons at 7–14 days in vitro (DIV).

### Fluorescence imaging

Neurons grown on the coverslip were incubated in Hank’s balanced salt solution (HBSS) containing 1 µM of FluoZin-3 AM, Fluo-2 AM, or DAF-FM for 30 min at 37℃. The cells were then washed with HBSS 3 times and placed in the recording chamber on the stage of an inverted microscope (Eclipse Ti-E, Nikon Inc., Japan) for fluorescence recording. The fluorescence indicators were excited with the light provided by a DG4 system (Sutter Instrument Co., Novato, CA, USA) through an excitation filter centered at 485 nm; the emitted light (510–540 nm) was captured through a 20× objective lens and directed to an ORCA Flash CMOS camera (Hamamatsu Photonics Inc., Japan). The recording system was controlled by NIS Element AR software (Nikon Inc., Japan) with an acquisition frequency of 1 s.

### Cell viability assay

According to the manufacturer’s instructions, we detect oxidoreductase activity in mitochondria with an MTT Assay kit. Neurons (1 × 10^6^ cells/ml) were cultured in a 24-well plate at 400 µl per well. After the glutamate treatment, the neurons were washed with the HBSS buffer and incubated in the neuro-basal medium containing MTT (0.4 mg/ml) for 1 h at 37 ℃. The reaction was stopped by adding SDS-HCl (10%). The enzyme activity and the background were calculated from the absorbance at 540 nm and 650 nm, respectively.

### Immunostaining

The neurons were fixed with 3.7% formaldehyde for 20 min and permeabilized with 0.05% Triton X-100 for another 20 min in a phosphate-buffered solution (PBS). The neurons were incubated in a PBS containing 3% bovine serum albumin for at least 30 min. Then, the neurons were incubated in a PBS containing anti-NLRP3 (#29125, Signalway Antibody, Inc., MA, USA) with 1:150 dilutions at 4℃ overnight. The next day, the neurons were washed with PBS and stained with goat anti-rabbit IgG (H + L) cross-adsorbed secondary antibody, Alexa Fluor™ 488 (1:200 dilutions, #A-11008 RRID: AB_143165) for 30 min at room temperature. Subsequently, the neurons were stained with 4’,6-diamidino-2-phenylindole (DAPI) for 20 min. Images were captured using a fluorescence microscope (Eclipse Ti, Nikon) with a 60× objective and confocal laser microscopy (SP5, Leica) with a 63× objective.

### Western blot assay

The cells in the 6-well plate were lysed with Radio-immunoprecipitation assay buffer (150 mM NaCl, 1.0% NP-40, 0.5% sodium deoxycholate, 0.1%SDS, 50 mM Tris, pH 7.5). After centrifugation (16,000 g, 30 min) the supernatant was collected the total protein lysate and the protein concentrations were determined by the bicinchoninic acid assay (Invitrogen, Waltham, USA.).For each sample, 30 µg was mixed with a Gel loading buffer (6×; 0.5 M Tris-HCl, pH 6.8, 30% glycerol, 10% SDS, 0.012% bromophenol blue, 0.6 M 1,4-Dithiothreitol and heated for 5 to 10 min at 100 ℃. The total protein samples were then separated by a 10% SDS-PAGE and transferred to a polyvinyl-difluoride membrane (Millipore, Billerica, MA, USA.). The membranes were then incubated in a tris-buffered saline (TBS) containing 3% BSA (bovine serum albumin) and 0.05% Tween-20 (TBST) for 1 h. After washing with TBS, the membranes were incubated in TBS containing different primary antibodies: anti-nNOS polyclonal antibody (61-7000, 1:1000 dilution, Thermo Fisher Scientific Inc., Waltham, USA), anti-phosphorylated nNOS^S847^ antibody (ab16650, 1:1000 dilution, Abcam, Cambridge, UK.), anti-phosphorylated nNOS^S1417^ antibody (ab5583, 1:1000 dilution, Abcam, Cambridge, UK.), and β-actin ((C4): sc-47778, 1:100 dilution, Santa Cruz Biotechnology, Inc., California, USA.) overnight at 4℃. After washing out the primary antibodies with TBST, the membranes were incubated with different secondary antibodies conjugated with horseradish peroxidase enzyme: anti-rabbit IgG (GTX213110-01, 1:5000 dilutions, GeneTex, USA) and anti-mouse IgG (GTX213111-01, 1:5000 dilutions, GeneTex, USA) for 1 h at room temperature. The membranes were developed with the ECL (enhanced chemiluminescence) system, and the proteins were detected using UVP ChemStudio PLUS Touch (Analytik Jena GmbH, Germany). The band intensities were quantified by using ImageJ software (National Institutes of Health, Bethesda, MD, USA, RRID: SCR_00370) and normalized according to the intensities of β-actin level.

### Data analysis

Unless noted otherwise, all experiments were repeated at least from 3 batches of cultured neurons. Curve fitting and statistical analysis were performed in imaging experiments using the Origin 2023b software (Origin Lab Corp., Northampton, MA). For the fluorescence imaging experiments of Ca^2+^, NO, and Zn^2+^, intensity traces for each neuron were normalized after background subtraction. The maximum change in the normalized trace was then identified to reflect the stimulation-induced response (Fig. S1). The data presented were the Mean ± SD, and no test for outliers was conducted. After passing the normality test, the significance was analyzed using a one-way ANOVA with the Tukey *post hoc* test. Differences were considered statistically significant at a *p*-value < 0.05. The statistical reports of each figure were described in the first section of the Supplementary Information.

## Electronic supplementary material

Below is the link to the electronic supplementary material.

Supplementary Material 1.

## Data Availability

All data generated or analysed during this study are included in this published article (and its Supplementary Information files).
