# Supplementary material for: Glutamate gradually elevates [Zn2+]i via the CaM–CaMKII–NOS cascade in primary cultured rat embryonic cortical neurons
Source: Sci Rep. 2025 Apr 30;15:15205. doi: 10.1038/s41598-025-99142-1 (PMC12043812; doi:10.1038/s41598-025-99142-1)
Supplement: Supplementary file 1 — Supplementary Material 1. [file 41598_2025_99142_MOESM1_ESM.docx]

Supplementary Material

Glutamate Gradually Elevates [Zn^2+^]_i_ via the CaM−CaMKII−NOS Cascade in Primary Cultured Rat Embryonic Cortical Neurons

Hui-Chiun Tseng^1^, Yong-Sheng Wang^1^, Chien-Yuan Pan^1,2*^

^1^ Department of Life Science, National Taiwan University, Taipei, Taiwan

^2^ Graduate Institute of Brain and Mind Science, National Taiwan University, Taipei, Taiwan

*** Correspondence:**Corresponding Author: [cypan@ntu.edu.tw](mailto:cypan@ntu.edu.tw)

1 Roosevelt Rd. Sec 4, Taipei 106, Taiwan, Tel: 886-2-33662452, E-mail: cypan@ntu.edu.tw

The ORCID ID numbers are 0000-0002-3047-6099 (HCT) and 0000-0002-6654-9309 (CYP)

**Keywords: calmodulin, calmodulin-dependent protein kinase II, inflammation, ionotropic glutamate receptor, nitric oxide synthase, Zn^2+^**

Statistical report of each figure:

Fig. 2b F value = 19.69799, N = 9, t(df) = 4 for each comparison

*p* value for each set of comparison

|  | Mock | Glu | AMPA | NMDA |
| --- | --- | --- | --- | --- |
| Glu | 1.05596E-9 |  |  |  |
| AMPA | 5.37055E-5 | 0.04191 |  |  |
| NMDA | 1.5233E-4 | 0.01826 | 0.99731 |  |
| AMPA+NMDA | 1.60593E-7 | 0.82198 | 0.35352 | 0.20312 |

Fig 2d:

F value = 12.17185, N =4, t(df) = 6 for each comparison

*p* value for each set of comparison

|  | Mock | Glu | AP5 | AP5+  Glu | DNQX | DNQX  +Glu |
| --- | --- | --- | --- | --- | --- | --- |
| Glu | 1.96726E-5 |  |  |  |  |  |
| AP5 | 1 | 2.03146E-5 |  |  |  |  |
| AP5+Glu | 0.16604 | 0.00729 | 0.17051 |  |  |  |
| DNQX | 1 | 2.3255E-5 | 1 | 0.19034 |  |  |
| DNQX+Glu | 0.99728 | 6.37349E-5 | 0.99813 | 0.39205 | 0.99908 |  |
| AP5+DNQX  +Glu | 0.92424 | 2.16934E-4 | 0.92867 | 0.71744 | 0.94547 | 0.9976 |

Fig.2f

F value = 29.37139, N =4, t(df) = 4

|  | Mock | Glu | DHPG | 4R-APDC |
| --- | --- | --- | --- | --- |
| Glu | 2.86737E-6 |  |  |  |
| DHPG | 1 | 3.00123E-6 |  |  |
| 4R-APDC | 0.99994 | 3.45352E-6 | 0.99998 |  |
| L-AP4 | 1 | 3.12124E-6 | 1 | 0.99999 |

Fig 3b

F value = 48.58848, N = 4, t(df) = 6 for each comparison

*p* value for each set of comparison

|  | Mock | Glu | W7 | W7+  Glu | KN62 | KN62  +Glu |
| --- | --- | --- | --- | --- | --- | --- |
| Glu | 638192E-8 |  |  |  |  |  |
| W7 | 1 | 6.45505E-8 |  |  |  |  |
| W7+Glu | 0.33914 | 5.13284E-8 | 0.41823 |  |  |  |
| KN62 | 1 | 6.35491E-8 | 0.99999 | 0.31208 |  |  |
| KN62+Glu | 0.00145 | 4.1167E-7 | 0.00205 | 0.16229 | 0.00128 |  |
| W7+KN62+Glu | 0.9149 | 6.97171E-8 | 0.95524 | 0.92869 | 0.89556 | 0.01829 |

Fig 3d

F value = 15.08868, N =5, t(df) = 6 for each comparison

*p* value for each set of comparison

|  | Mock | Glu | NIO | NIO+  Glu | NPA | NPA  +Glu |
| --- | --- | --- | --- | --- | --- | --- |
| Glu | 1.63542E-6 |  |  |  |  |  |
| NIO | 1 | 1.29203E-6 |  |  |  |  |
| NIO+Glu | 0.01032 | 0.03415 | 0.00813 |  |  |  |
| NPA | 1 | 1.34342E-6 | 1 | 0.00846 |  |  |
| NPA+Glu | 0.014 | 0.02558 | 0.01107 | 1 | 0.01151 |  |
| NIO+NPA  +Glu | 0.043 | 0.00802 | 0.03457 | 0.99643 | 0.03586 | 0.99902 |

Fig 4b

F value = 8.77412, N = 4, t(df) = 4 for each comparison

P value for each set of comparison

|  | Mock | Glu | AMPA | NMDA |
| --- | --- | --- | --- | --- |
| Glu | 0.00666 |  |  |  |
| AMPA | 0.00319 | 0.99509 |  |  |
| NMDA | 0.03903 | 0.88559 | 0.69655 |  |
| AMPA+NMDA | 5.54591E-4 | 0.69123 | 0.88196 | 0.22328 |

Fig 4d

F value = 6.37236, N =4, t(df) = 6 for each comparison

*p* value for each set of comparison

|  | Mock | Glu | W7 | W7+  Glu | KN62 | KN62  +Glu |
| --- | --- | --- | --- | --- | --- | --- |
| Glu | 0.00379 |  |  |  |  |  |
| W7 | 0.98381 | 6.26408E-4 |  |  |  |  |
| W7+Glu | 1 | 0.00473 | 0.97107 |  |  |  |
| KN62 | 1 | 0.00511 | 0.96541 | 1 |  |  |
| KN62+Glu | 0.90869 | 0.04685 | 0.48671 | 0.93816 | 0.94664 |  |
| W7+KN62+Glu | 0.9955 | 9.24287E-4 | 1 | 0.99017 | 0.98753 | 0.58976 |

Fig 4f

F value = 13.12325, N =4, t(df) = 6 for each comparison

*p* value for each set of comparison

|  | Mock | Glu | NIO | NIO+  Glu | NPA | NPA  +Glu |
| --- | --- | --- | --- | --- | --- | --- |
| Glu | 0.0001 |  |  |  |  |  |
| NIO | 0.94496 | 0.0001 |  |  |  |  |
| NIO+Glu | 0.1261 | 0.0018 | 0.58113 |  |  |  |
| NPA | 0.99806 | 0.0001 | 0.9988 | 0.31023 |  |  |
| NPA+Glu | 0.02047 | 0.01343 | 0.15957 | 0.97085 | 0.06273 |  |
| NIO+NPA+Glu | 0.46495 | 2.77101E-4 | 0.95968 | 0.97996 | 0.77821 | 0.61928 |

Fig 5b

F value = 50.08772, N =3, t(df) = 2 for each comparison

*p* value for each set of comparison

|  | Mock | Glu |
| --- | --- | --- |
| Glu | 6.8125E-4 |  |
| High K | 1.95239E-4 | 0.21789 |

Fig 5d

F value = 4.71057, N =7, t(df) = 2 for each comparison

*p* value for each set of comparison

|  | Mock | Glu |
| --- | --- | --- |
| Glu | 0.03202 |  |
| High K | 0.96696 | 0.05266 |

Fig 5f

F value = 10.70992, N =4, t(df) = 2 for each comparison

*p* value for each set of comparison

|  | Mock | Glu |
| --- | --- | --- |
| Glu | 0.00412 |  |
| High K | 0.53162 | 0.02148 |

Fig 6b

**For the nNOS^S847^**

F value = 0.82241, N =4, t(df) = 2 for each comparison

*p* value for each set of comparison

|  | Mock | Glu 15 min |
| --- | --- | --- |
| Glu 15 min | 0.61083 |  |
| Glu 30 min | 0.47696 | 0.96994 |

**For the nNOS^S1417^**

F value = 5.23375, N =4, t(df) = 2 for each comparison

*p* value for each set of comparison

|  | Mock | Glu 15 min |
| --- | --- | --- |
| Glu 15 min | 0.04066 |  |
| Glu 30 min | 0.96251 | 0.06136 |

Fig 7b

F value = 3.87514, N =6, t(df) = 3 for each comparison

*p* value for each set of comparison

|  | Mock | Glu | TPEN |
| --- | --- | --- | --- |
| Glu | 0.03246 |  |  |
| TPEN | 0.82105 | 0.17516 |  |
| TPEN+Glu | 0.9993 | 0.04204 | 0.87792 |

**Fig. S1. Quantification of Zn^2+^ imaging.** (a) A representative fluorescent image of neurons at the beginning of the recording. The numbers indicate regions of interest (circles, ROIs) selected for fluorescence intensity analysis. Scale bar: 10 μm. (b) Maximum changes in normalized fluorescence intensity for individual neurons. HBSS containing 100 μM glutamate was added to the chamber at the 60^th^ second, as indicated by the arrow (E). After background subtraction, the mean fluorescence intensity of each ROI before glutamate stimulation was defined as F_0_, and fluorescence intensities were normalized to their respective F_0_ values as F/F_0_ for each trace. The maximum F/F_0_ values after glutamate stimulation, minus the baseline, were defined as ΔF/ F_0_ for each neuron, such as the double arrows for traces 3 and 5.

**Fig. S2.** **TPEN suppresses the glutamate-induced elevation of [Zn^2+^]_i_ in cultured neurons.** We loaded the neurons with FluoZin-3 and added glutamate (100 μM) into the recording chamber with or without TPEN (0.4 μM). (a) The representative normalized average of fluorescence traces from one batch of the neurons. The arrow indicates the application of glutamate. The number of neurons was 30 for each trace. (b) The average changes in the normalized fluorescence intensities. The data presented were Mean ± SD (N = 4 batches of neurons). *: *p* < 0.05, ***: *p* < 0.001 when analyzed with one-way ANOVA with Tukey *post hoc* test.

F value = 23.72309, N =4, t(df) = 3 for each comparison

*p* value for each set of comparison

|  | Mock | Glu | TPEN |
| --- | --- | --- | --- |
| Glu | 5.04039E-5 |  |  |
| TPEN | 0.99958 | 5.80293E-5 |  |
| TPEN+Glu | 0.02018 | 0.01199 | 0.0242 |

**Fig. S3** High-frequency depolarization of a patched neuron elevates [Zn^2+^]_i_ in nearby neurons. Neurons were loaded with FluoZin-3, and whole-cell patch-clamp recordings were performed in HBSS at room temperature using an EPC-10 amplifier controlled by the Pulse program (v8.7; HEKA Elektronik, Germany). The pipette solution contained (in mM): 120 aspartic acid, 5 MgCl₂, 40 HEPES, 0.1 EGTA, 2 ATP, and 0.3 GTP, adjusted to pH 7.3 with KOH (310 mOsm). The membrane potential was held at -70 mV and depolarized to +30 mV for 5 ms at 100 Hz for 1 minute. (a) Bright-field image of neurons. A neuron was patched using a glass micropipette, and the numbers on the soma indicate neurons selected for fluorescence recording. (b) Normalized fluorescence traces. "Break-in" marks the establishment of the whole-cell patch, and the solid bar (Depol.) indicates the period of depolarization. The dashed line represents F/F_0_ = 1. (c) The ionic current of the patched neuron during high-frequency depolarization. The inset displays five depolarization recorded at the 5th second.

**Fig. S4. Agonists of glutamate receptors elevate the [Ca^2+^]_i_ in cultured neurons.** We loaded neurons with Fluo2, a Ca^2+^-sensitive fluorescence dye, and monitored the [Ca^2+^]_i_ represented by normalized fluorescence intensities (F/F_0_). Neurons were stimulated with glutamate, AMPA, NMDA, AMPA+NMDA, DHPG, APDC, and L-AP4 (100 μM each). (a. & c) Representative averaged F/F_0_ from one batch of neurons. SD was displayed at the last data point, and the arrows indicate the chemicals used. (b & d) Changes in F/F_0_ (ΔF/F_0_). * and ***: Student's *t*-test with *p* < 0.05 and 0.001, respectively, compared with the Mock group.

Fig. S4b F value = 19.81578, N = 9, t(df) = 4 for each comparison

P value for each set of comparison

|  | Mock | Glu | AMPA | NMDA |
| --- | --- | --- | --- | --- |
| Glu | 1.79901E-7 |  |  |  |
| AMPA | 1.66017E-7 | 1 |  |  |
| NMDA | 1.23617E-4 | 0.24594 | 0.23393 |  |
| AMPA+NMDA | 2.89272E-8 | 0.98397 | 0.98698 | 0.08684 |

Fig. S4d

F value = 42.2731, N =5, t(df) = 4

|  | Mock | Glu | DHPG | 4R-APDC |
| --- | --- | --- | --- | --- |
| Glu | 0.0001 |  |  |  |
| DHPG | 0.0218 | 0.0001 |  |  |
| 4R-APDC | 0.98514 | 0.0001 | 0.00706 |  |
| L-AP4 | 0.98348 | 0.0001 | 0.06539 | 0.83416 |

**Fig. S5. TPEN did not inhibit glutamate-induced NO generation in cultured neurons.** We loaded neurons with NO indicator, DAF-FM (5 μM), and stimulated them with glutamate (100 μM) in the presence or absence of TPEN (0.4 μM). (a) Representative fluorescence traces from one batch of neurons. The data presented were Mean with SD displayed at the last time point. The arrow indicates the application of glutamate. (b) The average glutamate-induced changes (ΔF/F_0_). Data were reported as the Means ± SD and analyzed using one-way ANOVA with the Tukey *post hoc* test (*: *p* < 0.05 compared with the Mock group or as indicated).

F value = 4.52549, N =5, t(df) = 3 for each comparison

*p* value for each set of comparison

|  | Mock | Glu | TPEN |
| --- | --- | --- | --- |
| Glu | 0.02834 |  |  |
| TPEN | 0.50444 | 0.34112 |  |
| TPEN+Glu | 0.03297 | 0.99983 | 0.3787 |

**Fig. S6. High-K⁺ stimulation increases nNOS phosphorylation at Ser^1417^ in 30 min.** We stimulated cultured neurons with a High-K^+^ buffer and collected total protein at 15 and 30 minutes for Western blot analysis using specific antibodies against nNOS, Ser^847^-nNOS, and Ser^1417^-nNOS. For each condition (Mock, 15 min, and 30 min), three dishes were harvested from the same neuronal culture preparation. Band intensities of phosphorylated nNOS were first normalized to total nNOS and then to the average of the Mock group. (a) Representative immunoblots from triplicate samples. (b) Quantification of normalized staining levels. Band intensities of Ser^847^ and Ser^1417^ were normalized to the average of their respective Mock group. Data are presented as mean ± SD (n = 3).

F value = 7.40145, N =3, t(df) = 2 for each comparison

*p* value for each set of comparison

|  | Mock_Ser^1417^ | Ser1417_15 min |
| --- | --- | --- |
| Ser1417_15 min | 1 |  |
| Ser1417_30min | 0.00489 | 0.00489 |

**Fig. S7. Glutamate does not affect the function of mitochondria in 30 min in cultured cortical neurons.** We stimulated the neurons with glutamate (100 μM) for 15 and 30 min, both in the absence and presence of TPEN (0.4 μM), with subsequent monitoring of cell viability by MTT assay. The data were Mean ± SD derived from 4 independent experimental sets. Significance was assessed via one-way ANOVA, followed by Tukey *post hoc* test, with statistical significance determined at *p* < 0.05.

**Fig. S8 Long-term glutamate treatment reduce viability in cultured neurons.** We treated the cultured neurons with 30 and 100 μM of glutamate for 6 (a) and 18 (b) hrs. We then use MTT assay to monitor the cell viability. The data were Mean ± SD derived from 4 independent experimental sets. Significance was assessed via one-way ANOVA, followed by Tukey *post hoc* test; ***: *p* < 0.001.

6 hr

F value = 4.86291, N = 4, t(df) = 3 for each comparison

*p* value for each set of comparison

|  | Mock | 30 μM |
| --- | --- | --- |
| 30 μM | 0.99685 |  |
| 100 μM | 0.06139 | 0.0543 |

18 hr

F value = 47.7908, N = 4, t(df) = 3 for each comparison

*p* value for each set of comparison

|  | Mock | 30 μM |
| --- | --- | --- |
| 30 μM | <0.0001 |  |
| 100 μM | <0.001 | 0.43633 |


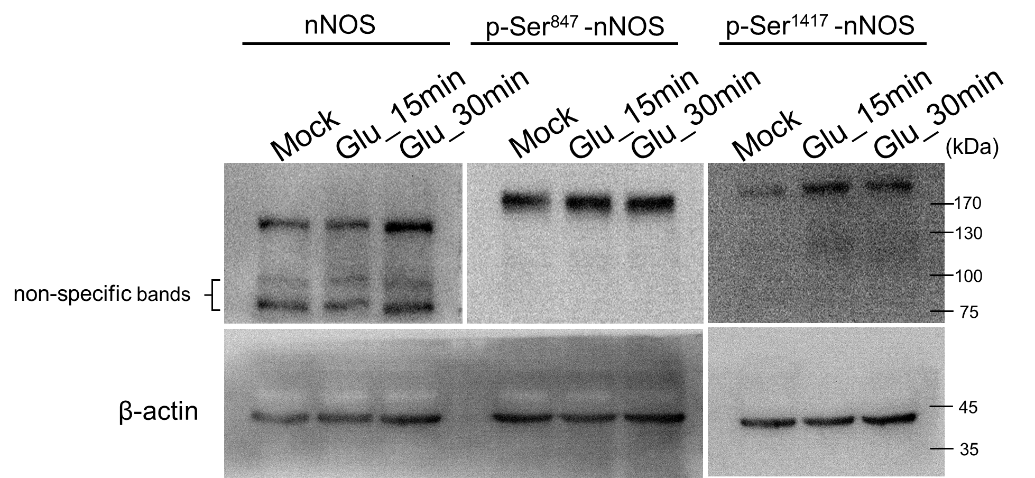


**Fig S9 Original blot images shown in Fig. 6**. Cell lysates isolated from cultured neurons treated with buffer (Mock) and glutamate (100 μM) for 15, and 30 min were loaded into the wells of a polyacrylamide gel as indicated. After transblotting, the membrane was cut into 5 pieces for staining with different antibodies as indicated.
